# Water scarcity can be a critical limitation for the poultry industry

**DOI:** 10.1007/s11250-023-03599-z

**Published:** 2023-05-22

**Authors:** Mohamed I. El Sabry, Zeinab U. Romeih, Farid K. R. Stino, Abeer R. Khosht, Samul E. Aggrey

**Affiliations:** 1 Faculty of Agriculture, Cairo University, 6 El-Gamma Street, Giza, 12613 Egypt; 2 Animal Production Research Institute, Agricultural Research Center, 9 Nadi Al-Sayed St, Giza, Egypt; 3 Department of Poultry Science, University of Georgia, Athens, GA USA

**Keywords:** Climate change, Water shortage, Water footprint, Water restriction, Water conversion ratio, Genetic selection, Meat quality, Layers

## Abstract

Water is essential for various physiological functions and the productive performance of animals. However, with climatic uncertainties exacerbated by climatic changes, water could become a scarce nutrient shortly. This is already the case in one-third of the world’s countries, which are under medium to high levels of water stress. Accordingly, with the growth of poultry production, the availability of water at ad libitum level may not be guaranteed, and birds can be under water restriction for variable periods. Thus, this article aims at attracting the attention of animal scientists to the freshwater shortage challenge, as well as shedding light on (1) the effects of climate change on the freshwater resources; (2) the effects of limited access to water, either by water restriction (WR) or water deprivation (WD), on the growth, feed efficiency, and meat quality of broilers; (3) the effects of different levels of WR or WD on egg production and egg quality traits; (4) the effects of limited access to water on the health, behavior, and welfare status of chickens; and (5) suggested solutions to overcome future water shortage challenges. In conclusion, severe water shortage/restriction might negatively influence the productivity, behavior, and welfare status of the chickens. Genetic background and environmental conditions may interact with the WR effects. The tolerance level of indigenous chicken breeds to limited water access could provide the knowhow to potential solutions to overcome water shortage problems. Selection of chicken strains with high tolerance capacity to thirst and limited water access regimens may be a sustainable solution for solving water scarcity problems.

## Introduction

Poultry is one of the main sources of animal protein due to its universal acceptability, high nutritional value, and health benefits. The growth of the global population (7.8 billion) has pressured the poultry industry to increase its capacity (Marangoni et al. 2015; El Sabry et al. 2018a; 2022). From 1980 to 2004, global meat production doubled and it is projected to be doubled again from 2000 to 2050 (FAO, 2005; Steinfeld et al. 2006). This fast growth of global meat production poses pressure on water resources, because livestock production is a very water-intensive agricultural activity; i.e., about one-third of the total water that is utilized in global agricultural production is assigned to animal production. Water footprint (WF) is a water metric measurement that has been used to accurately calculate water use in relation to final products. According to Mekonnen and Hoekstra (2010; 2012), the WF per kg of meat for beef cattle, sheep, pig, goat, and chicken are 15,400, 10,400, 6000, 5500, and 4300 L of water, respectively. Thus, WF can be utilized as an efficient tool for sustainable freshwater management in livestock production.

Climate change has created new challenges such as increasing the earth’s temperature by 0.2 °C per decade with significant fluctuation in the amount and distribution of rainfall. Thus, heat waves and water scarcity can affect the future of poultry production, health, and welfare (El Sabry et al. 2021a, c; Abbas et al. 2022; Morgado et al. 2022).

**Fig. 1:**
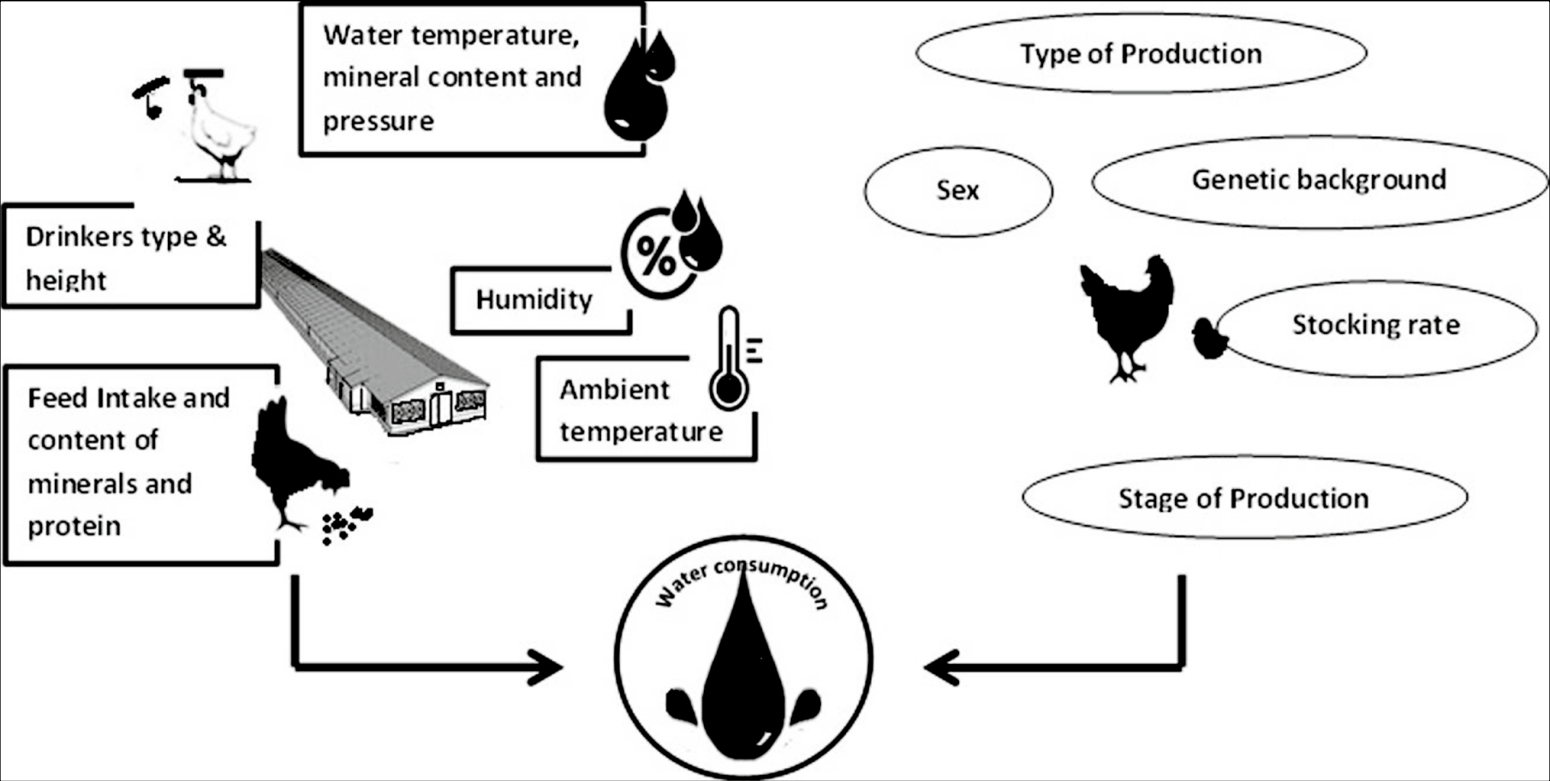
Factors affecting water consumption in chickens

Several factors affect the daily water requirement for poultry: e.g., housing conditions (temperature, light regimen and intensity, etc.), performance level, and feeding-related factors (type and ingredients). Also, Xin et al. (1994) showed that age affects water consumption and based on that developed an equation to predict daily water consumption of broiler chicken between 1 and 56 days of age: daily water use (DWU) for 1000 birds per day =  − 2.78 + 4.70D + 0.128D^2^ − 0.00217D^3^, where D is the age of birds in days. Some of the factors that can affect water consumption in poultry are summarized in Fig. 1.

The implications of water scarcity may be a future challenge that can hinder the development of poultry production at both industry and backyard levels especially in arid and semi-arid regions. Water shortages or restrictions could also be a limitation in achieving food security in some regions of the world.

## Effect of climate change on freshwater resources

Water scarcity refers to freshwater demand exceeding availability (Savenije, 2000; Kummu et al. 2016). Freshwater is 3% of the world’s water, but only 0.5% is useable. Agriculture uses about 72% of the freshwater, while other activities (municipalities for households and industries) use 28% of water (UN-Water, 2021). Water scarcity has raised a major global issue that limits people’s life quality and agriculture development, even in countries with adequate water resources (UNICEF, 2018; 2021). However, water scarcity mainly affects the people in rural areas; e.g., around 3.2 billion people live in water-stressed agricultural regions (FAO, 2012; 2020).

Several factors have contributed to freshwater scarcity, including the growth of the global population, urbanization, water pollution, and/or the poor management of water resources. However, climate change is the main reason that threatens the sustainability of freshwater resources (Arnell et al., 2011; UNICEF, 2021; Leal Filho et al. 2022). For instance, the Mediterranean basin has experienced higher temperatures than the average and alterations in precipitation rates, which have adversily impacted its water budget and increased the frequency of droughts (Dai, 2013; Cramer et al. 2018).

Water scarcity is a significant pbroblem in many regions of the world. Based on the map published by the UN-WATER report in 2021, it is clear that north Africa and most of western and central Asia suffer from critical water stress (freshwater withdrawal as a proportion of available freshwater resources > 100%). While the Indian subcontinent and the Republic of South Africa suffer from medium water stress (50–75%), China, Germany, France, Spain, and the USA suffer from low water stress (25–50%) (UN-Water, 2021), which is the current situation. By 2025, two-thirds of the world’s population may face water shortages (Brar et al. 2022).

## The effect of limited access to drinking water on chickens

Water is a fundamental nutrient to maintain the productive and reproductive performances, health, and welfare status of birds. Therefore, water should be available at an acceptable quality and in sufficient amounts (El Sabry et al. 2018b; 2021a). Limiting access to drinking water can be applied in two ways: water restriction (WR, controlling the water amount of ad libtium water intake) or water deprivation (WD, controlling the water supply for a certain period of time).

### The effects of limited access to drinking water on broilers performance

#### Productive traits

The relationships between feed intake (FI), water intake (WI), and feed conversion ratio (FCR) are well established (Bierer et al., 1966b; Marks, 1981). Reduction in water accessibility would result in a reduction in feeding activity (Abdelsamie and Yadiwilo, 1981; Leeson et al., 1997; 2000). However, the genetic background can affect the response of broilers to limited access to water. Marks (1981) observed that the WI of fast-growing broilers was greater than that of slow-growing ones when feed and water were available ad libitum and the FCR of fast-growing birds was more impaired than that of slow-growing strains when subjected to WR. In addition, Kellerup et al. (1965) observed that WR levels of 10, 20, 30, 40, and 50% of ad libtum affected the performance of crossbred broilers. The FI and body weight (BW) decreased with increased WR levels up to 50%, while FCR was impaired when the WR level reached 20%. Thus, these findings indicate that WI plays a role in enabling genetic growth potential.

Environmental conditions also can affect hydration stress. Under thermo-neutral conditions, Viola et al. (2009) applied different WR levels (10, 20, 30, and 40% of ad libtum) from day 1 to day 21 of age. They reported a significant linear reduction in daily FI and body weight gain (BWG), and a worse FCR with increasing WR level. Under a tropical environment, Kese and Baffour-Awuah (1982) reported that increasing WR levels of 15, 30, and 45% of ad libitum WI from 1 to 8 weeks of age impaired the BWG and FCR of broilers. Also, Abdelsamie and Yadiwilo (1981) applied different levels of WR over a 9-week period and reported a significant depression in BWG and worse FCR throughout the studied period.

In most tropical regions, slow-growing indigenous chicken breeds are well-adapted to harsh weather conditions and resistant to diseases, e.g., Naked-neck (NNK), Ovambo (OVB), and Fayoumi chickens (El Sabry et al. 2012; Chikumba et al. 2014). Chikumba et al. (2014) investigated the response of NNK and OVB chickens to water stress. They found a decline in BWG, FI, WI, and slaughter weight (16 weeks of age) associated with an increase in the WR levels, 40% and 70% of the ad libitum amount, but the OVB chickens had a superior performance (BWG, WI) and slaughter weight at 16 weeks.

Toghyani et al. (2011) also reported that WD for 6 h followed by 2 h of water-access, from 7 to 17 days of age decreased the BW and FCR from 17 to 46 days of age thereby suggesting that poor FCR of water-deprived chicks may be due to regurgitating feed and water after supplying water. However, they observed that WD for 12 h significantly decreased FI during the period from 17 to 28 days of age only, but not from 7 to 17 days of age.

Also, Miller et al. (1988) reported that FI, WI, and BW declined in a cyclic watering scheme of broilers older than 21 days (15 min each 3-h cycles). However, when the watering cycle was 30 min every 4 h, no decrease in performance was observed. 

Also, Ndlela et al. (2019) also exposed broilers to different WD periods and demonstrated that increasing the length of the WD period (6, 8, 12, and 24 h) impaired BWG, FI, and FCR for broilers of 14–25 days of age. But from 25 to 36 days of age, less negative effects on the productive performance were observed. These results indicated that WD for any of the abovementioned periods reduces growth performance, but broiler chickens may adapt to hydric stress as they advance in age (more than 25 days).

To answer whether compensatory water behavior does exist, Viola et al. (2005) studied the effects of WR levels (0, 10, 20, 30, and 40% of ad libtum WI) on broilers until 21 days of age, then thereafter received water ad libitum from 22 to 28 days of age. The compensatory water consumption was calculated as the difference in WI between birds subjected and not subjected to WR. The results indicated that, at all levels of WR, there was a compensatory water consumption, mainly during the first days of the ad libitum period. Water intake decreased as the birds adapted to the new condition. Higher WI was observed in 30 and 40% WR groups than in 10% WR group. Also, Kapkowska and Gerry (1995) showed that applying the WD regimen (water available 15 min/ three times at 07:00, 12:00, and 17:00 h per day for a week) during the early stage of age (1–3 weeks of age) significantly decreased the BW, which was compensated after supplying water. They also reported that applying this WD regimen from 3 to 6 weeks of age did not affect the BW of chicks. Contrarily, Toghyani et al. (2011) reported that at 28–46 days of age, water-deprived chicks showed a slight increase in FI but WD decreased the BW by 8% at day 28 of age and by 5% at slaughter age (46 days) compared to the group that had unlimited access to water. They concluded that chicks are unable to fully compensate for the initial loss of the BWG during the deprivation period and ensuing periods in subsequent ad libitum water access times.

There is a contradiction about the effect of WD on the mortality rate. Bierer et al. (1966b) observed that the survival time was similar in groups deprived of water, feed, or feed and water. The average survival time for 1-day and 7-day-old chicks subjected to a full WD was 5 and 7 days, respectively. They also observed that survival rate increased with the advance in age. Kese and Baffour-Awuah (1982) reported that WR of about 15–45% of ad libitum WI for the entire growing period did not significantly affect the mortality rate, but WR significantly affected the growth performance, which agrees with the findings of Kellerup et al. (1965) and Viola et al. (2009).

#### Meat quality parameters

Understanding the effects of limited access to water on meat quality characteristics is crucial not only for consumer preferences, but also for evaluatinig chicken strains, which may be drought-resilient (higher water utilization efficiency). Chikumba et al. (2013) and Iheukwumere and Herbert (2003) reported that WR could change the blood constituent profile, e.g., triglycerides, cholesterol, total protein, albumin, and globulin concentrations in the serum of water-restricted chicks. Tissue and organ depletion because of water restriction in broilers was associated with elevated enzyme activities of alkaline phosphatase (ALP), alanine transaminase (ALT) and aspartate transaminase (AST) (Fasina et al., 1999). Chikumba et al. (2014) and Ndlela et al. (2019) reported that severe WR (40% and 70% WR of ad libitum WI) or WD (6–12–18 24 h) decreased muscle fat content and thickness of breast meat cuts. These alterations can be due to the depletion of glycogen reserves and a high rate of catabolism of fat.

Ndlela et al. (2019) found that at 24 h post-slaughter, the breast meat of water-deprived chicks (6, 12, 18, or 24 h) became paler. Also, Chikumba et al. (2014) reported that redness (a*) value of meat from NNK chickens that were exposed to 40% of ad libitum WI was higher than that of meat from a control (free access to water) group.

Chikumba et al. (2014), Üstuner (2014), and Ndlela et al. (2019) showed that some meat quality parameters were not affected by limited access to water, e.g., pH, water holding capacity (WHC), ash, and protein content of the breast meat in different indigenous and commercial chicken breeds.

### Laying hens productivity and egg quality

In White Leghorns, an intermittent watering regimen showed a consistent improvement in feed efficiency and BW (Spiller et al. 1976). However, Savory (1978) found that restricting the daily water supply to 90% of the ad libitum level for 6 weeks resulted in a decline in FI. Similarly, Fujita et al. (2001) found that restricting water to only 20% of ad libtium WI (500 ml/day) for 7 days significantly reduced daily FI in 6-month-old commercial laying hens, compared to those receiving 200 ml or 300 ml water/day.

Under hot and dry conditions in Saudi Arabia of 37.2–38.6 °C and 20–37 RH%, Ahmed and Alamer (2011) studied the effect of 2 weeks of WR (20 and 40% of ad libitum WI) on 50-week-old commercial layers and local chicken breeds. They reported that WR did not clearly affect the FI; however, FI of commercial layers tended to be less than that of local breeds. In addition, WR of 40% of ad libitum WI decreased egg production % in both commercial strains and local breeds.

Also, Bierer et al. (1965) reported that the average survival time was 6 days for water-deprived (24 h/day) White Leghorn hens at 40 weeks old, under the ambient temperature of 29 °C and ad libitum feed. Also, Bierer et al. (1966a) reported that after 30 days of WD treatment under 14 °C ambient temperature, 1-year-old White Leghorn laying hens survived up to 13 days, while non-laying hens survived up to 23 days. Adams (1973), on the other hand, observed that short-term WD (48–72 h) increased the mortality rate. It appears that laying hens have a better chance at survival with WD when compared to broiler chickens.

The effect of WD on the BW and productivity of layers was investigated by Arad (1982). He compared the effect of WD for 48 h and heat stress (up to 44 °C) on BW loss in a pure local Egyptian Sinai breed, a commercial layer strain (Leghorn), and two crosses (Sinai × Leghorn and Leghorn × Sinai). They reported that the relative weight loss in the commercial Leghorn breed was the highest compared to the other breeds and compensatory BW gain was the lowest for the Leghorn breed.

It was suggested that the severity of water limitation on egg production may be correlated with age and/or stage of production of the birds. Fujita et al. (2001) found that WR to 100 ml/day of ad libtium WI (500 ml/day) for 7 days markedly reduced the egg production rate in 6-month-old commercial Shaver Starcross 288 Layers.

Bierer et al. (1966a) reported that White Leghorn hens that received feed but no water reduced their egg production after 3 days of WD. After 24 and 48 h of WD, the eggshell quality declined, and the incidence of cracked eggs increased. Also, 24 h of WD slightly reduced egg production, while 48 and 72 h of WD severely decreased egg production (Wilson and Edwards, 1952). Similar results were observed when 50-week-old White Leghorn laying hens were subjected to WD for 48 to 72 h (Adams 1973).

Summers and Leeson (1976) described that accidental WD for 48 h resulted in a drastic short-term effect on egg number; egg production declined by 4% within 6 days, remained at this level for 7 days, and then returned to the previous level 14 days later. During the WD period, the Haugh unit (albumen height) of eggs from water-deprived hens was higher compared to those of eggs from hens of the control group. Water-deprived birds produced eggs with significantly lower eggshell deformation values. A period of WD, alone, may provide a rest period, which is sufficient to induce subsequent improvements in egg quality parameters without necessitating a prolonged unproductive period (Summers and Leeson 1976).

Finally, in old layers, Bierer et al. (1965), Adams (1973), and Summers and Leeson (1976) reported that WD resulted in a reduced egg production and smaller eggs with thin-shelled eggs compared to their counterparts without water deprivation.

### Effects of limited access to water on the health of chickens

 The adverse effects of WR on the immunological traits of chickens have been confirmed by several studies. In broilers, Toghyani et al. (2011) showed that WD (2 or 12-h period/day) at an early age (from day 7 to day 17 of age) decreased the heterophil to lymphocyte ratios and antibody titers against Newcastle disease and sheep red blood cells (SRBC). Similarly, in laying hens, Ahmed and Alamer (2011) indicated that short-term WR (40% of ad libtum WI) for 2 weeks reduced the SRBC antibody titer in a local breed but not commercial strain.

Wilson and Edwards (1952), Bierer et al. (1965), Bierer et al. (1966a), and Bierer et al. (1966a; 1966b) found that limited water access can cause lesions such as nephrosis, gizzard erosion, greenish gizzard contents, visceral gout, and congestion and ulceration of the lower intestine. These signs may be due to the lack of feed and/or WI.

Iheukwumere and Herbert (2003) evaluated the white blood cells (WBC; leukocyte counts) in water-restricted broiler chicks. They noted that there is a significant relationship between WBC count and severe WR levels. In two indigenous chicken breeds, Chikumba et al. (2013) showed that breeds respond differently to WR, and that Naked Neck (NNK) birds had higher WBC values (*P* < 0.05) than Ovambo (OVB) birds at 40% water restriction level, but lower WBC than OVB at 70% WR level.

### Effects of limited access to water on behavior and welfare aspects of chickens

 In the last two decades, the term “animal welfare” has been increasingly used by animal scientists, corporations, veterinarians, politicians, and consumers. However, this term means different things to each category of people. Hewson (2003) stated that animal welfare depends on the health condtion and feelings of the animal, and environmental conditions. Animal’s feelings evolve to respond to stimulants (Duncan, 2002). Thus, in 2008, the World Organization for Animal Health considered an animal to be in a good welfare state if it is healthy, comfortable, well-nourished, expressing species-specific behavior, and not suffering from distress, fear, and/or pain (World Organization of Animal Health, 2008). A feelings-based approach to welfare research typically measures behavioral outcomes and signs due to exposing the animals to stress or abnormal conditions (Hewson, 2003). Thus, an improper environment could lead the animals to express abnormal behavior, which can be repetitive actions that are fixed in form and orientation and serve no purpose (Savory, 1995).

Thirst is a subjective perception that motivates an animal to drink as it is a sensation aroused by a lack of water and associated with a desire to drink fluids (Adams et al. 2020), which impacts animal welfare (Vanhonacker et al. 2008; Tuyttens et al. 2010). Providing the optimal requirements of good quality water is necessary for maintaining chicken performance and the gut health and morphology (El Sabry and Abd El-Ghany 2021; El Sabry et al., 2018a). An adult broiler chicken usually drinks about 150 to 200 ml per day, and it can reach up to 300–350 ml per day (Tabler, 2003; Appleby et al., 2004). Limiting water access to drinkers would negatively affect their welfare status and productive performance.

In broilers, WR changes the drinking and feeding behavior of chickens (Viola et al. 2005, 2009). Viola et al. (2009) subjected chicks from 1-day old to 21 days of age to WR up to 40% (10, 20, 30, and 40% of ad libitum WI). They reported that broilers showed abnormal behavior, e.g., stopped eating, became excited, running, and jumping into the cages. When water was re-provided to birds, some chicks showed aggressive behavior, e.g., pecking the toes of others, and began drinking very fast (Viola et al. 2005; 2009). Broilers exposed to WR (10, 20, 30, and 40% of ad libitum WI) after being provided with water ad libitum, showed compensatory water consumption, initially taking large bouts of water before settling down to normal drinking behavior (Viola et al. 2005).

Boone et al. (2009) studied the effect of interaction between WD (6, 12, and 24 h) and familiarity of chicks with drinkers on the drinking behavior. They reported that chicks that were familiar with drinkers started to drink early compared to those that were unfamiliar with the drinkers. Water-deprived chicks also drank for longer periods than those in the control group after 6 h, 12 h, and 24 h of WD. Preening behavior, which is a comfort behavior, was more prevalent in the groups of chickens familiar with drinkers than those in the unfamiliar group. Additionally, compared to the 24 h deprived groups, the hens with unlimited access to water displayed noticeably higher stretching behavior. A higher frequency of exploratory activity toward the drinker was also observed in the hens of the 24-h deprivation group, particularly in those who were familiar with the drinker.

The effect of WD on times of unconsciousness and death was investigated. It was concluded that WD had no impact on the latency to unconsciousness but altered the process by increasing the time to death for broilers at 22, 36, and 50 days of age (Leeson et al., 2007; Viola et al., 2009; Vanderhasselt et al. 2013; Baker-Cook et al. 2021). Therefore, free access to water is vital for animal welfare requirements (Vanhonacker et al., 2008; Tuyttens et al., 2010; Rault et al., 2016).

In laying hens, Rault et al. (2016) tested the effect of various durations of WD (12, 18, 24, or 32 h) on the behavior of layers using a motivation test, which is based on passing through a narrow vertical gap (by changing its width; 150, 135, 120, or 100 mm) to access the water side of the testing cage. They reported that the hens’ willingness to pass through a narrow vertical gap to access the water of chicks of all WD groups was similar. However, hens changed their behavior as early as 12 h after WD, the first time point, while from 24 to 32 h of WD a plateau was reached in terms of behavioral adaptation. According to Toghyani et al. (2011), WR had no effect on walking ability, tibial dyschondroplasia, foot pad, hock burn, or valgus/varus angulation.

## Suggested solutions for overcoming water shortage problem

Solving the water shortage issue of the poultry industry can be either direct or indirect by reducing water loss or by enhancing the water utilization efficacy of birds. Thus, in the following sections, we suggest some applications that may attract the attention of multidisciplinary work groups to unearth methods for mitigating the water shortage problem in water-stressed regions and suggesting future management plans for water usage in agricultural activities.

### Breeding programs

Breeding programs have been suggested as a sustainable tool mitigating some of the climatic changes. Chikumba et al. (2014) and El Sabry et al. (2021b) suggested that breeding programs for developing heat-stress-resistant chicken breeds should be established. Similarily, water-efficient chickens can be developed.

### Improving water utilization capacity of poultry facilities

Water supply equipment as well as drinkers and nipples could be designed to minimize leakage and water spills to reduce water loss in chicken production. There seems to be some utility in controlled water deprivation. However, such a strategy requires more detailed research. The effects of application of magnetized water are unclear, however, it has been suggested that conditioning water with a strong magnet alters the physiochemical properties of drinking water, which subsequently affects the absorption and the utilization of soluble minerals in the drinking water. Some investigations have shown that it can enhance the productivity and the quality of poultry. For example, it can increase the breaking strength of the eggshell, egg number, and semen quality (El Sabry et al., 2018b; 2021a; 2022).

## Conclusions

Water restriction ≥ 10% of ad libtium WI or water deprivation for ≥ 6 h/day impairs broiler performance. Moreover, water restrcion ≥ 40% of ad libtium WI or water deprivation for ≥ 6 h/day negatively influences some meat quality paramaters. Limited access to water alters feeding, and locomotor behavior in broilers. However, it is noteworthy that broiler chicks from 1 to 25 days of age are more sensitive to limited water access than chicks that are older than 25 days.

Laying hens showed better endurance to water deprivation compared to broiler chickens. However, either water restriction to 100 ml/day of ad libtium WI (500 ml/day) for 7 days markedly reduced the egg production rate. In addition, water deprivation for 24 h slightly decreases egg production, while water deprivation for ≥ 48 h markedly decreased egg production and egg quality.

Performance and behavior responses of broiler breeds (indigenous, crossbred, and commercial) to limited access to water are different. Thus, breeding programs for selecting high water efficacy strains and watering regimens can be a plausible sustainable solutions for solving water shortage problems.

## Data Availability

N/A.

## Code availability

N/A.
